# Supplementary material for: Association between anxiety and non-coding genetic variants of the galanin neuropeptide
Source: PLoS One. 2019 Dec 27;14(12):e0226228. doi: 10.1371/journal.pone.0226228 (PMC6934320; doi:10.1371/journal.pone.0226228)
Supplement: Attachment — Submitted filename: Manuscript Keszler.docx [file pone.0226228.s001.docx]

The manuscript "Association between anxiety and non-coding genetic variants of the galanin neuropeptide" by Gergely Keszler and coworkers studies the genetic association of six non-coding single nucleotide variations in the GAL gene with anxiety and depression in healthy young students (mean age 22 years). From their results the authors conclude that only allele T of the rs 10442577 SNP and association of the rs948854C-rs4432027 allele combination are associated with greater lebels of anxiety. Neither association was obseved of other SNP in the GAL gene and depression. The experiments were carried out in an adeguate manner, respect the ethics or publication ethics, and the documentation of the results is satisfactory. Overall, the manuscript is nicely written, satisfies the PLOS ONE criteria for pubblication and is worth publishing. However, I have several comments about the choice of population and the results obtained from this study. The results aren't very interesting. The association between SNP in the GAL genes and anxiety is very low. It is very difficult to demonstrate this association in healthy young people. The works you cited show a strong correlation between SNPs of genes for GAL in anxious subjects, with major depression, panic disorders and multiple schlerosis. As you described in the conclusions, these differences could be ascribed to the fact that in those works they recruit patients with different psychiatric pathologies. You have excluded subjects with psychiatric history from your samples, but it would have been more interesting to study the polymorphisms for the GAL genes in these subjects or in people with family histories of psychiatric illnesses, to try to study the susceptibility of these people for anxiety, depression and other psychiatric disease
