# Supplementary material for: Association between anxiety and non-coding genetic variants of the galanin neuropeptide
Source: PLoS One. 2019 Dec 27;14(12):e0226228. doi: 10.1371/journal.pone.0226228 (PMC6934320; doi:10.1371/journal.pone.0226228)
Supplement: Attachment — Submitted filename: Response to reviewers.docx [file pone.0226228.s002.docx]

**Response to the Academic Editor**

Dear Dr. De Luca, Budapest, 16 October 2019

Thank you very much for encouraging us to review and improve our manuscript (PONE-D-19-16922) according to your and the reviewers’ suggestion. During the revision of the manuscript, every point of criticism and suggestion of the reviewers has been adressed and the new version has been revamped accordingly. Please find beneath both response letters to the reviewers.

Sincerely yours,

on behalf of all co-authors,

Gergely Keszler

corresponding author

**Response to Reviewers**

Fisrt of all, we would like to thank both anonymous Reviewers for thoroughly revising our paper, appreciating its merits and raising valuable concerns which prompted us to rethink several points and to amend the manuscript accordingly. Please find beneath our point-by-point response to your comments and criticism.

Importantly, a common major concern of both Reviewers was whether it is relevant to study association of GAL polymorphisms with anxiety and depression on a normal, healthy young population rather than on a clinical sample. We admit it should have been better explained in the original manuscript, and are grateful to you for having drawn our attention to our negligence.

Quotations from reviewers’ comments are written in italics, our responses are typed in normal letters and revised sections of the manuscript are highlighted bold.

*Reviewer #2:*

*1. It is very difficult to demonstrate this association in healthy young people. The works you cited show a strong correlation between SNPs of genes for GAL in anxious subjects, with major depression, panic disorders and multiple schlerosis. As you described in the conclusions, these differences could be ascribed to the fact that in those works they recruit patients with different psychiatric pathologies. You have excluded subjects with psychiatric history from your samples, but it would have been more interesting to study the polymorphisms for the GAL genes in these subjects or in people with family histories of psychiatric illnesses, to try to study the susceptibility of these people for anxiety, depression and other psychiatric disease*

As cited in the Introduction of the manuscript, numerous studies found firm genetic association between GAL gene polymorphisms and depression, anxiety, alcohol dependence and panic disorder in psychiatric in- or outpatients. In our study, however, we have intentionally chosen a non-clinical, healthy population to extend the findings of others, that is, to study whether the effect of GAL polymorphisms could be observed on the mood characteristics of a normal cohort. Notably, the anxiogenic effect of a single SNP proved to be significant even in this context, and this effect has never been described before even on a clinical population. This prompts us to check whether the rs1042577 T allele and the rs948854_C_rs4432027_C haplotype as predilection factors are enriched in a clinical sample with anxiety too.

**Psychiatric disorders are known to have a strong polygenic genetic background and a lot of environmental stressors (life events) might precipitate the development of symptoms. We chose a young healthy population as the prevalence of negative life events (such as getting divorced, unemployed, widowed, bereaved, having low socio-economic status or afflicted by severe diseases) is far less among these people, making the genetic components more exposed and identifiable. On the other hand, our randomly selected non-clinical population is genetically heterogeneous, i.e. represents a continuous spectrum of mood dimensions, and might comprise subjects with milder (subclinical) forms of anxiety and depression** such as GAD (generalized anxiety disorder) or PDD (persistent depressive disorder) as indicated by higher HADS scores on the corresponding scales. **Recent studies conducted on healthy samples also found that HADS anxiety and depression scores vary a lot among university students (Andrews et al., 2006; Kebede et al., 2019; Gan and Yuen Ling, 2019).**

To emphasize the above considerations, the bold-marked text above was incorporated in the Discussion and the following paragraph has been inserted in the Introduction:

**“As described above, other authors have already demonstrated associations between GAL polymorphisms and mood disorders, but no studies have been done to date to test this possible association in a non-clinical, normal healthy sample. Thus, our goal was to figure out whether any genetic association is detectable between a set of marker SNPs representing the entire regulatory landscape of the galanin gene and mood characteristics on the normal spectrum as well.”**

*2. The results aren't very interesting. The association between SNP in the GAL genes and anxiety is very low. It is very difficult to demonstrate this association in healthy young people.*

The Reviewer is right in saying that differences between the HADS scores of cohorts with different genotypes are quite low. However, due to the polygenic nature of psychological traits, a single non-coding polymorphism being even in candidate genes typically does not account for greater effects. For comparison, we refer to a paper by de Moor et al. (2012) who performed a meta-analysis on genome-wide association studies for personality traits on a 17,375-strong sample. Despite that large sample size, the variance did not exceed 0.2%. It is of note that in a previous genetic association study (Kótyuk et al., 2013) performed on polymorphisms of the GDNF gene we have got differences commensurable to those in the present manuscript which also proved statistically significant.

As far as the enrollment of healthy, young people is concerned, we refer to the explanation above.

*Reviewer #1*

*1. My concerns are about the nature of the subjects studied vis-à-vis issues of anxiety and depression as psychiatric disorders, to which the authors give detailed attention, and the rating scale used to develop a “phenotype” (subjects with greater or lesser amounts of anxiety and/or depression on the HADS).*

*Concerning the HADS (ref 31 in the MS), it is a “self-assessment scale…found to be a reliable instrument for detecting states of depression and anxiety in the setting of an hospital medical outpatient clinic. The anxiety and depressive subscales are also valid measures of severity of the emotional disorder… The research was conducted in general medical outpatient clinics on adults of both sexes between the ages of 16 and 65 who suffered from a wide variety of complaints and illnesses.” The HADS thus was not designed to measure anxiety or depression in a sample of normal young adult college students with no complaints or current illnesses.*

The Hospital Anxiety and Depression Score (HADS), a psychometric tool was originally developed and has since been widely used to detect depression and anxiety in patients with physical health problems (Zigmond and Snaith, 1983). Over the subsequent decades the test has attained an established role in quantitative psychometrics with almost 5,000 scientific references to date, and it also underwent several re-evaluations and its scope was extended several times.

By reviewing the literature in 2002, Bjelland et al. concluded that it is an adequate tool to assess mood characteristics in the general population too. One of the founding fathers of the test proposed that the questionnaire was valid when used in community settings too (Snaith, 2003). Finally, the intriguing question whether the HADS could measure anxiety and depression in healthy subjects was addressed by Caci et co-workers (2003). Their landmark study concluded that the HADS inquiry battery is sensitive enough to discriminate between two levels of anxiety in a non-clinical, healthy population. They identified these constructs as ‘anxiety’ and ‘restlessness’ (inner tension), the latter interpreted as low-intensity anxiety with lower scores on the HADS-A scale. Their work has paved the way for dozens of recent analyses deploying HADS to quantitate anxiety and depression in normal healthy subjects, especially in cohorts of university students from all over the world with statistically firm data and conclusions (Andrews et al., 2006; Andrews and Wilding, 2004; Gan and Yuen Ling, 2019; Kebede et al., 2019; Lee et al., 2016; Liu et al., 2009; Moreira de Sousa et al., 2018; Sadeghi et al., 2019; Silva and Figueiredo-Braga, 2018). As it is based on a healthy cohort, our manuscript blends well in this series of investigations, being one of a few recent genetic association studies using HADS scores in healthy populations (Jiménez et al., 2018; Loja-Chango et al., 2016; Kotyuk et al., 2013). The latter is actually a former project of ours that analyzed the same healthy population with HADS to set up associations between non-coding genetic polymorphisms in the GDNF gene and anxiety. Thus, the current manuscript is a direct corollary of that one.

To briefly reflect the above, the following paragraph has been incorporated to the Phenotype characteristics section of the manuscript: “**The HADS questionnaire measures anxiety and depression on 7-7 items, scoring on a 0 to 3 Likert scale. It was originally developed to assess anxiety and depression in case of non-psychiatric hospital patients [31]. However, a great deal of studies have shown that it is an adequate tool to measure mood characteristics on non-clinical samples as well (Caci et al., 2003).**”

2. *The mean anxiety score (6.55 +/- 3.7 [which I assume is the standard deviation – not stated]) is in the lowest group of scores (Table 1 in ref 31), which consisted of 98% non-and doubtful-cases of clinical anxiety. As well, the mean depression score (2.80 +/- 2.7) was in the lowest group of scores (Table1 in ref 31), which again consisted of 98-99% non-and doubtful-cases of clinical depression. In short, the normal subjects’ scores were clustered near the low end of the HADS, providing little variance for correlational analysis with the genetics data – not surprising for the use of a hospital clinic-derived scale on normal subjects with no complaints or illnesses. More appropriate depression and anxiety scales should have been used to develop the subjects’ “phenotype.”*

Yes, in scores like 6.55 ± 3.7, the ± value denotes the standard deviation. Thank you for your comment, we indicated it in every table of the manuscript.

Well, it is obviously true that the mean scores on the two subscales are near the lower end of the scale in our sample – especially in case of the depression subscale, which is ‘normal’ considering the nature of the data. However, in the context of our sample size and using the same statistical toolbox which most studies did as quoted above – especially ANOVA that focuses on the variability of the dependent variable - , we got statistically significant correlation data even after Bonferroni’s correction for multiple setting. While maximally respecting the opinion of the Reviewer, we take the liberty of hinting that one might not directly compare the HADS scores and cut-off values from the initial study of Zigmond and Snaith conducted on a clinical sample back in 1983 to those measured in our normal, healthy Central European population that differs from theirs in so many respects.

*3. Given the use of an inappropriate rating scale on normal subjects, the lengthy discussions in the Introduction and Discussion sections of the relationship of galanin to psychiatric illnesses is of little relevance. I suggest the authors severely shorten the MS and present it as straightforward correlative study between galanin gene-related SNPs and a psychometric measure of anxiety and depression in normal individuals with no psychiatric illness. The authors should provide one or two sentences as rationale for performing the study, limiting any comment about galanin and psychiatric illness to a short, suggestive statement about its relationship to classical neurotransmitters and that it has been found to be altered in certain psychiatric illnesses, and including the list of references.*

As it is a psychiatric genetic study addressing both molecular geneticists, psychologists and psychiatrists, we deemed it important to review and discuss the available literature on galanin and its polymorphisms in sufficient detail to make it understandable for all readers. However, accepting the argumentation of the Reviewer, we have streamlined the Introduction as you suggested regarding correlation between galanin and psychiatric illnesses (see the revised version of the manuscript).

As far as the shortening of the Discussion is concerned, we assume it is important to compare our findings observed in our healthy population to those obtained in transcriptional studies, cell and animal models as well as people with psychiatric problems in order to provide our Readers a comprehensive insight into the relevance of our data in the broader context too. In our opinion, cutting parts of the Discussion would impair the integrity of the paper to a large extent, so we decided not to modify it.

**References**

# Andrews B, Hejdenberg J, Wilding J. [Student anxiety and depression: comparison of questionnaire and interview assessments.](https://www.ncbi.nlm.nih.gov/pubmed/16764939) J Affect Disord. 2006 Oct;95(1-3):29-34. Epub 2006 Jun 9.

# Andrews B, Wilding JM. [The relation of depression and anxiety to life-stress and achievement in students.](https://www.ncbi.nlm.nih.gov/pubmed/15527535) Br J Psychol. 2004 Nov;95(Pt 4):509-21.

# [Bjelland I](https://www.ncbi.nlm.nih.gov/pubmed/?term=Bjelland%20I%5BAuthor%5D&cauthor=true&cauthor_uid=11832252), [Dahl AA](https://www.ncbi.nlm.nih.gov/pubmed/?term=Dahl%20AA%5BAuthor%5D&cauthor=true&cauthor_uid=11832252), [Haug TT](https://www.ncbi.nlm.nih.gov/pubmed/?term=Haug%20TT%5BAuthor%5D&cauthor=true&cauthor_uid=11832252), [Neckelmann D](https://www.ncbi.nlm.nih.gov/pubmed/?term=Neckelmann%20D%5BAuthor%5D&cauthor=true&cauthor_uid=11832252). The validity of the Hospital Anxiety and Depression Scale. An updated literature review. [J Psychosom Res.](https://www.ncbi.nlm.nih.gov/pubmed/?term=Bjelland%2C+Dahl%2C+Haug%2C) 2002 Feb;52(2):69-77.

# Caci H, Baylé FJ, Mattei V, Dossios C, Robert P, Boyer P. [How does the Hospital and Anxiety and Depression Scale measure anxiety and depression in healthy subjects?](https://www.ncbi.nlm.nih.gov/pubmed/12759165) Psychiatry Res. 2003 May 1;118(1):89-99.

de Moor MH, Costa PT, Terracciano A, Krueger RF, de Geus EJ, Toshiko T, Penninx BW, Esko T, Madden PA, Derringer J, Amin N, Willemsen G, Hottenga JJ, Distel MA, Uda M, Sanna S, Spinhoven P, Hartman CA, Sullivan P, Realo A, Allik J, Heath AC, Pergadia ML, Agrawal A, Lin P, Grucza R, Nutile T, Ciullo M, Rujescu D, Giegling I, Konte B, Widen E, Cousminer DL, Eriksson JG, Palotie A, Peltonen L, Luciano M, Tenesa A, Davies G, Lopez LM, Hansell NK, Medland SE, Ferrucci L, Schlessinger D, Montgomery GW, Wright MJ, Aulchenko YS, Janssens AC, Oostra BA, Metspalu A, Abecasis GR, Deary IJ, Räikkönen K, Bierut LJ, Martin NG, van Duijn CM, Boomsma DI. [Meta-analysis of genome-wide association studies for personality.](https://www.ncbi.nlm.nih.gov/pubmed/21173776) Mol Psychiatry. 2012 Mar;17(3):337-49. doi: 10.1038/mp.2010.128. Epub 2010 Dec 21.

Gan GG, Yuen Ling H. [Anxiety, depression and quality of life of medical students in Malaysia.](https://www.ncbi.nlm.nih.gov/pubmed/30846664) Med J Malaysia. 2019 Feb;74(1):57-61.

Jiménez KM, Pereira-Morales AJ, Adan A, Lopez-Leon S, Forero DA. [Depressive symptoms are associated with a functional polymorphism in a miR-433 binding site in the FGF20 gene.](https://www.ncbi.nlm.nih.gov/pubmed/30241547) Mol Brain. 2018 Sep 21;11(1):53. doi: 10.1186/s13041-018-0397-0.

Kebede MA, Anbessie B, Ayano G. [Prevalence and predictors of depression and anxiety among medical students in Addis Ababa, Ethiopia.](https://www.ncbi.nlm.nih.gov/pubmed/31080499) Int J Ment Health Syst. 2019 May 6;13:30. doi: 10.1186/s13033-019-0287-6. eCollection 2019.

Kotyuk E, Keszler G, Nemeth N, Ronai Z, Sasvari-Szekely M, Szekely A. [Glial cell line-derived neurotrophic factor (GDNF) as a novel candidate gene of anxiety.](https://www.ncbi.nlm.nih.gov/pubmed/24324616) PLoS One. 2013 Dec 6;8(12):e80613. doi: 10.1371/journal.pone.0080613. eCollection 2013.

Lee SJ, Park CS, Kim BJ, Lee CS, Cha B, Lee YJ, Soh M, Park JA, Young PS, Song EH. [Association between morningness and resilience in Korean college students.](https://www.ncbi.nlm.nih.gov/pubmed/27572002) Chronobiol Int. 2016;33(10):1391-1399. Epub 2016 Aug 30.

Liu Q, Shono M, Kitamura T. [Psychological well-being, depression, and anxiety in Japanese university students.](https://www.ncbi.nlm.nih.gov/pubmed/19263468) Depress Anxiety. 2009;26(8):E99-105. doi: 10.1002/da.20455.

Loja-Chango R, Pérez-López FR, Simoncini T, Escobar GS, Chedraui P. [Increased mood symptoms in postmenopausal women related to the polymorphism rs743572 of the CYP17 A1 gene.](https://www.ncbi.nlm.nih.gov/pubmed/27117436) Gynecol Endocrinol. 2016 Oct;32(10):827-830. Epub 2016 Apr 27.

Moreira de Sousa J, Moreira CA, Telles-Correia D. [Anxiety, Depression and Academic Performance: A Study Amongst Portuguese Medical Students Versus Non-Medical Students.](https://www.ncbi.nlm.nih.gov/pubmed/30332368) Acta Med Port. 2018 Sep 28;31(9):454-462. doi: 10.20344/amp.9996. Epub 2018 Sep 28.

Sadeghi O, Keshteli AH, Afshar H, Esmaillzadeh A, Adibi P. [Adherence to Mediterranean dietary pattern is inversely associated with depression, anxiety and psychological distress.](https://www.ncbi.nlm.nih.gov/pubmed/31185883) Nutr Neurosci. 2019 Jun 11:1-12. doi: 10.1080/1028415X.2019.1620425.

Silva RG, Figueiredo-Braga M. [Evaluation of the relationships among happiness, stress, anxiety, and depression in pharmacy students.](https://www.ncbi.nlm.nih.gov/pubmed/30236427) Curr Pharm Teach Learn. 2018 Jul;10(7):903-910. doi: 10.1016/j.cptl.2018.04.002.

Snaith RP. [The Hospital Anxiety And Depression Scale.](https://www.ncbi.nlm.nih.gov/pubmed/12914662) Health Qual Life Outcomes. 2003 Aug 1;1:29.

Zigmond AS, Snaith RP. [The hospital anxiety and depression scale.](https://www.ncbi.nlm.nih.gov/pubmed/6880820) Acta Psychiatr Scand. 1983 Jun;67(6):361-70.
