# Supplementary material for: Association between anxiety and non-coding genetic variants of the galanin neuropeptide
Source: PLoS One. 2019 Dec 27;14(12):e0226228. doi: 10.1371/journal.pone.0226228 (PMC6934320; doi:10.1371/journal.pone.0226228)
Supplement: Attachment — Submitted filename: Answers To Reviewer 1.docx [file pone.0226228.s003.docx]

**Authors’ response to Reviewer #1**

Dear Professor Rubin,

Thank you very much for your careful re-evaluation of our revised manuscript. We are very grateful for your valuable suggestions on how to substantiate our largely speculative allegations in the Discussion.

1. We totally agree with your remarks on the use of the HADS questionnaire in a seemingly normal sample of students recruited for this study. To emphasize better that our study differs in this respect from other studies using clinical samples for association studies, the following sentence has been inserted into the introductory section of the manuscript: (association was tested) “*in an ostensibly normal sample consisting of university students with no psychiatric history”*, and it is highlighted again in the second sentence the Discussion chapter: “*In contrast to former research aimed to find genetic associations between GAL gene variants and mood characteristics in subjects with clinical anxiety and depression, the novelty of the present study is that it was performed in a cohort of apparently normal, healthy volunteers.*”

2. To expel your concerns regarding our speculative assumption on the potentially marked stratification of our (seemingly) normal sample (students without psychiatric history), we tested whether the HADS scores of the top 10% cohorts of students reach the clinical range on the depression and anxiety subscales – as you suggested.

As described in the ‘Phenotype characteristics’ paragraph of the ‘Materials and methods’ section, the mean anxiety score in the total sample was 6.55 (± 3.7 standard deviation) and the mean depression score was 2.80 (± 2.7). Upon setting up the “top 10%” and “bottom 10%” cohorts, it turned out that 11.4% of the sample (n=60 individuals) scored lower than 3 and 9.9% of the sample (n=52) scored higher than 11 on the anxiety scale. 9.7% of the sample (n=51) scored higher than 6 and 11.6% of students (n=61) scored 0 on the depression scale.

Descriptive statistics for these cohorts are as follows. **Anxiety** mean score ± standard deviation for the anxiety bottom 11.4%: 1.32 ± 0.673; and for the **top 9.9%: 14.23 ± 2.278.** Depression mean score ± standard deviation for the **depression** bottom 11.6%: 0 (all depression scores were 0 for all members of this cohort); and for the **top 9.7%: 9.160 ± 1.902**. Hermann et al. (1995) and Caci et al. (2003) found that the cut-off values for “abnormal”, i.e. clinical depression and anxiety were 11 and 9 HADS scores, respectively. These data corroborate our assumption that a significant portion of our healthy, non-clinical sample had clinical anxiety and depression indeed.

These findings prompted us to perform genetic association analyses on these cohorts as well. Results of these tests can be seen in the table beneath. The Chi square analysis of allele frequencies in the top versus bottom 10% cohorts revealed statistically significant association of the rs1042577 SNP with anxiety and depression. However, the association between GAL rs948854 and rs4432027 with anxiety was not observable in this context, possibly due to low cohort sizes.

| **dbSNP  number** |  | **Anxiety** | | | **Depression** | | |
| --- | --- | --- | --- | --- | --- | --- | --- |
|  |  | **low- anxiety cohort (n=60)** | **high- anxiety cohort (n=52)** | **P** | **low- depression cohort (n=61)** | **High- depression scores (n=51)** | **p** |
| rs948854 | C | 31.4% | 39.4% | 0.209 | 36.7% | 39.2% | 0.696 |
|  | T | 68.6% | 60.6% |  | 63.3% | 60.8% |  |
| rs2097042 | C | 29.6% | 37.0% | 0.272 | 37.7% | 40.2% | 0.726 |
|  | T | 70.4% | 63.0% |  | 62.3% | 59.8% |  |
| rs4432027 | C | 30.2% | 39.6% | 0.151 | 36.8% | 39.1% | 0.736 |
|  | T | 69.8% | 60.4% |  | 63.2% | 60.9% |  |
| rs694066 | A | 14.4% | 14.7% | 0.950 | 10.2% | 15.2% | 0.270 |
|  | G | 85.6% | 85.3% |  | 89.8% | 84.8% |  |
| rs3136540 | T | 27.3% | 33.7% | 0.322 | 32.1% | 31.7% | 0.957 |
|  | C | 72.7% | 66.3% |  | 67.9% | 68.3% |  |
| **rs1042577** | **T** | **30.6%** | **50.0%** | **0.005** | **36.8%** | **51.2%** | **0.048** |
|  | **C** | **69.4%** | **50.0%** |  | **63.2%** | **48.8%** |  |

We decided to mention these interesting data in the Discussion section to provide further evidence for the association described between the rs1042577 T allele and anxiety from a different approach and to underpin our speculative sentences with statistical data. The Discussion has been shortened and tautened by removing non-relevant or lengthy speculative sentences, according to your suggestions.

Thank you again for your inspiring comments that made us rethink the concept by obtaining further data in support of the biological relevance of GAL polymorphism in anxiety and depression.

Sincerely yours,

Gergely Keszler

corresponding author
